# Enhancement of Bone-Marrow-Derived Mesenchymal Stem Cell Angiogenic Capacity by NPWT for a Combinatorial Therapy to Promote Wound Healing with Large Defect

**DOI:** 10.1155/2017/7920265

**Published:** 2017-01-24

**Authors:** Kangquan Shou, Yahui Niu, Xun Zheng, Zhanjun Ma, Chao Jian, Baiwen Qi, Xiang Hu, Aixi Yu

**Affiliations:** Department of Orthopedics, Zhongnan Hospital of Wuhan University, Wuhan, Hubei 430071, China

## Abstract

Poor viability of engrafted bone marrow mesenchymal stem cells (BMSCs) often hinders their application for wound healing, and the strategy of how to take full advantage of their angiogenic capacity within wounds still remains unclear. Negative pressure wound therapy (NPWT) has been demonstrated to be effective for enhancing wound healing, especially for the promotion of angiogenesis within wounds. Here we utilized combinatory strategy using the transplantation of BMSCs and NPWT to investigate whether this combinatory therapy could accelerate angiogenesis in wounds. In vitro, after 9-day culture, BMSCs proliferation significantly increased in NPWT group. Furthermore, NPWT induced their differentiation into the angiogenic related cells, which are indispensable for wound angiogenesis. In vivo, rat full-thickness cutaneous wounds treated with BMSCs combined with NPWT exhibited better viability of the cells and enhanced angiogenesis and maturation of functional blood vessels than did local BMSC injection or NPWT alone. Expression of angiogenesis markers (NG2, VEGF, CD31, and *α*-SMA) was upregulated in wounds treated with combined BMSCs with NPWT. Our data suggest that NPWT may act as an inductive role to enhance BMSCs angiogenic capacity and this combinatorial therapy may serve as a simple but efficient clinical solution for complex wounds with large defects.

## 1. Introduction

The healing of large areas of full-thickness skin defects is a challenging clinical problem [1, 2]. Optimum healing of a cutaneous wound requires an effective way to stimulate rapid angiogenesis because blood vessels are crucial for wound repair for delivering oxygen and nutrients to the host cells in the wounds [3]. More importantly, the formation of new blood vessels is fundamental to sustain the granulation formation, which is indispensable stage for wound healing [4].

Mesenchymal stem cells have been shown to play an important role in the healing of cutaneous wounds [5]. Ideally, after the dermal wounding, endogenous mesenchymal stem cells (MSC) will be mobilized into the circulation, homing into the wounded skin and eventually differentiating into skin cells [6]. Although extremity ischemia is a powerful stimulant for marrow stem cell recruitment, fewer progenitor cells were able to migrate to the ischemic wound [7]. As a result of that, allogeneic BM-MSCs derived from healthy donors have been used to treat diseases in humans [8]; however, their contribution to wound healing, especially their differentiation during the healing process, has not been fully understood. In addition, the poor viability and low levels of BMSCs engraftment often limit their therapeutic potential [9].

Currently, several reports have described the environmental factors, including physical forces and soluble factors, driving MSC differentiation toward endothelial cell line, which is helpful to angiogenesis [10]. Namely, their hypothesis was realized by creating shear force stimuli with exogenous growth factors such as vascular endothelial growth factor (VEGF), modifying MSCs to overexpress VEGF [11], or through addition of specific inhibitors, including simvastatin [12]. However, these strategies are considered as time-consuming and relative expensive [13].

Negative pressure wound therapy (NPWT) is a promising treatment that has become widely adopted and applied for wound repair since its advent over 15 years ago [14]. It can function as a therapy to draw the wound together and provide stimulating micromechanical forces [15]. It has been proven to induce a granulation tissue response [16] and enhance the microvascular circulation within the wound [17]. Skin defects could exhibit positive response to NPWT treatment leading to healing with or without skin graft [18]. However, whether the NPWT could serve as a inductive factor to stimulate MSCs behaviors such as angiogenesis capacity still remains to be identified.

Several studies reported the application of negative pressure to influence the biological behavior of cells in vitro. Wilkes et al. designed a bioreactor to explore the effect of subatmospheric pressure upon the fibroblasts and observed changed morphology, thicker appearance, and organized actin cytoskeleton of cells [19]. Previously, we have proved that the inductive effect of NPWT (−125 mmHg) on rat periosteum-derived mesenchymal stem cells osteogenic differentiation in a 3D fibrin matrix through a subatmospheric perfusion bioreactor [20]. However, to date, little is known about the effect of higher-intensity NPWT (e.g., −150 mmHg) on bone-marrow-derived mesenchymal stem cells (BMSCs) differentiation into the cutaneous wound healing related cell types and the benefit for the accelerated angiogenesis during wound healing. Considering that the mechanical forces exerted by NPWT can serve as an advantageous therapy to induce gradient level of hypoxia within the wounds and BMSCs may behave as a positive reaction under this hypoxia microenvironment [21, 22], the feasibility of combined strategy using NPWT and transplantation of BMSCs is worth evaluating. In this study, we assessed NPWT effects on BMSCs viability and differentiation behavior in vitro under NPWT. Furthermore, we investigated the effect of this combinatorial therapy and whether it could serve as an efficient way to accelerate angiogenesis in an animal cutaneous wound model.

We hypothesized that NPWT would improve the viability of the BMSCs and induce BMSCs differentiation into the cutaneous tissue related cell types and enhance their angiogenic capacity, leading to an accelerated angiogenesis for wound healing and regeneration.

## 2. Materials and Methods

### 2.1. BMSCs Isolation, Culture, and Characterization

All the experimental procedures were according to the guidelines for the Care and Use of Laboratory Animals of the National Institutes of Health and approved by the Institutional Animal Care and Use Committee (IACUC) of Wuhan University.

Briefly, 3-week-old male Sprague-Dawley (SD) rats (Laboratory Animal Center of Wuhan University, China) were sacrificed, and the bone marrow was obtained by flushing the marrow cavity with complete BMSC culture medium containing low-glucose Dulbecco's modified Eagle's medium (L-DMEM, Hyclone, USA) supplemented with 10% fetal bovine serum (FBS, Gibco, USA) and 1% penicillin-streptomycin (P/S, Invitrogen, USA). After centrifugation, the cells were resuspended and cultured at 37°C in 5% CO_2_. Cells of 3-4 passages were used for the following experiments. BMSCs were characterized by flow cytometry analysis at the third passage. CD90 (Biolegend, USA), CD45 (Biolegend, USA), CD44 (Biolegend, USA), and CD31 (Affymetrix eBioscience, USA) were used to identify the surface marker of BMSCs.

### 2.2. In Vitro NPWT Treatment

For the NPWT group, cells were cultured in the flask at a pressure of −150 mmHg for 9 days. Briefly, BMSCs were seeded in the T25 (25 cm^2^) at 37°C in 5% CO_2_ in incubator, and one needle was penetrated through the plug cap and inserted into the flask, and the end of the needle was connected to a vacuum pump (VSD Inc.) that generated continuously suction at continuous −150 mmHg. The control group was maintained in a similar incubator and same device but without negative pressure.

### 2.3. Assessment of the Morphology, Viability, and Proliferation of BMSCs under NPWT

BMSCs cultured in the flask under NPWT or not were fixed in 4% paraformaldehyde (Aspen, China) for 1.5 h and stained with FITC-conjugated phalloidin (Yeasen, China) for 1 h. Finally, samples were stained with diamidine-phenylindole-dihydrochloride (DAPI, Aspen, China) and analyzed with a confocal fluorescence microscope system (Leica SP2, Leica, Germany).

To evaluate cell proliferation under NPWT, on days 1, 3, 6, and 9 after NPWT treatment, a CCK-8 assay was performed. BMSCs cultured with normal pressure served as control. To assess BMSCs viability and death under NPWT, a live/dead assay was performed. On days 1, 3, 6, and 9, the cells in the culture flask were incubated with 1 mM calcein AM (Wako, Japan) for 1 h and then incubated with 1 ug/mL propidium iodide (PI, Invitrogen, USA) for 5 min at 37°C. Next, the cells were imaged using a fluorescence microscopy (IX51, Olympus, Japan). Live cells stained green, whereas dead cells stained red.

### 2.4. Full-Cutaneous Skin Wound Healing Model

Male SD rats weighing 250–300 g were purchased from the Laboratory Animal Center of Wuhan University (China) in accordance with the Institutional Animal Care and Use Committee (IACUC) of Wuhan University.

Rats were randomly divided into four groups: sham, NPWT group, local BMSC injection, or BMSC + NPWT group (*n* = 9 in each group). On day 0, animals were anesthetized with ketamine hydrochloride (60 mg/kg body weight), and 35 mm full-thickness excisional wounds (including panniculus carnosus) were created on the disinfected and shaved backs of the rats. For the BMSCs injection group, 2.5 × 10^6^ BMSCs were resuspended in 100 *μ*L PBS and injected intradermally at 12 equidistant points around the wound edge. For the NPWT group, the wounds were covered with a 3.5 cm × 3.5 cm area of Duoderm foam (VSD Medical Technology Co. Ltd, Wuhan, Hubei, China) and then covered with a Vacuum Assisted Closure Device with a constant negative pressure values at continuous −150 mmHg. The connecting tubes of VAC device were long enough to avoid affecting the ambulation or the routine life of animals such as eating and drinking. For the BMSC + NPWT group, the cells injection were the same as the BMSCs injection group, and then the NPWT were applied as described before. For the sham group, no treatment was performed except for generation of the full-thickness excisional wound. Finally, all of the wounds were covered with a semipermeable membrane (VSD Medical Technology Co, Ltd, Wuhan, Hubei, China) to prevent leakage and drying. All of the animals were housed individually. Using the same procedures, a new dressing was applied every 3 days in all groups.

On days 0, 3, 6, and 9 after operation, the wounds were photographed and wound tissues were harvested. For histological analysis, the harvested specimens were immediately snap-frozen in Optimal Cutting Temperature compound (OCT compound) (Sakura Finetek, Torrance, CA) or fixed in 4% paraformaldehyde overnight and embedded in paraffin for hematoxylin and eosin (H&E) staining and Masson's Trichrome staining and immunohistochemistry and immunofluorescence analyses.

The wound area was calculated using Image Pro Plus 6.0 (IPP 6.0) software (Media Cybernetics, USA). The calculation formula was performed as follows: (wound area on day 0 − wound area day “*X*”)/(wound area on day 0) × 100. On day 9, the formation of granulation tissue and wound maturity were assessed according to the reported scoring system based on the evaluation of the H&E and Masson's Trichrome staining results [23] (Table 1).

### 2.5. Real-Time Quantitative PCR

BMSCs were cultured under NPWT or normal condition for 9 days. For in vivo RT-PCR, wound tissues harvest at indicated time points was stored at −80°C. Then, Total RNA was extracted via the TaKaRa MiniBEST Universal RNA Extraction Kit (Takara Bio, Japan) following the manufacturer's instructions. cDNA was synthesized using the PrimeScript™ II First Strand cDNA Synthesis Kit according to the manufacturer's protocol (Takara Bio, Japan). PCR conditions comprised an initial step of denaturation for 1 min at 95°C, followed by total 40 cycles of 15 s at 95°C, 20 s at 58°C and 20 s at 72°C. After normalization against the housekeeping gene *β*-actin, the expression of genes of interest (shown in Table 2) was measured using the 2^−ΔΔCt^ method.

### 2.6. Western Blotting Analyses

Total protein was isolated from BMSCs cultured under NPWT or normal condition or from wound tissues harvested at the indicated time points with a Total Protein Extraction Kit (Aspen, China). Equal amounts of protein from cells or tissue lysates were loaded onto a 5% SDS polyacrylamide gel (Aspen, China) and transferred to a polyvinylidene fluoride (PVDF) membrane (Millipore, USA). Membranes were blocked with 5% BSA in TBS and then incubated with primary antibodies against CD31 (1 : 500), *α*-SMA (1 : 5000), VEGF (1 : 1000), NG2 (1 : 100), and *β*-actin (1 : 10,000 for cell lysates and 1 : 3000 for tissue lysates) for overnight at 4°C. Next, an HRP-conjugated secondary antibody was applied (1 : 10,000) and detected with the Immobilon Western Chemiluminescent HRP Substrate system (Millipore, USA). All of the antibodies were purchased from Abcam Inc (United Kingdom).

### 2.7. Immunofluorescence

BMSCs cultured under NPWT or normal condition or the wound tissues on day 9 were fixed in 4% paraformaldehyde for 20 min. For wound tissues, the samples were further embedded in paraffin and cross-sectioned at 5 *μ*m. Then, the samples were stained with primary antibodies against CD31 (1 : 100), *α*-SMA (1 : 100), VEGF (1 : 200), and NG2 (1 : 50). After incubation with Cy3-conjugated secondary antibodies (1 : 50, Aspen, China), samples were added with DAPI to stain the cell nuclei.

### 2.8. Enzyme-Linked Immunosorbent Assay (ELISA)

The concentrations of TGF-*β*_1_ in the wound lysates on day 9 were measured by using ELISA from a Rat LAP Kit (TGF beta 1, Ready-SET-Go, Affymetrix eBioscience, USA), according to the manufacturer's instructions.

### 2.9. Assessment of Wound Vascularization

To assess blood vessel formation, immunohistochemistry was performed on day 6. The tissue sections described above were incubated with primary antibodies (anti-collagen type IV, 1 : 100, and anti-CD31, 1 : 100, Abcam, United Kingdom) overnight at 4°C and then incubated with HRP-coupled secondary antibodies (Aspen, China). Staining was performed using diaminobenzidine (brown). For each section, tube-like structures were considered to be newly formed blood vessels and were quantified. Furthermore, to quantify the number of mature blood vessels, six sections from different tissue samples were selected from each treatment group for immunofluorescence analysis on day 9. CD31 (for endothelial cells), *α*-SMA (for smooth muscle cells), and cell nuclei were stained red, green, and blue, respectively. Red and green costaining was considered to represent mature blood vessels. All of the evaluation procedures were performed independently by two pathologists.

### 2.10. Statistical Analysis

All of the values are expressed as mean ± SD. Statistical significance between two groups was measured using the Student's unpaired *t*-test (two-tailed). The difference among different groups was detected using one-way analysis of variance (ANOVA) test. A *p* value < 0.05 was considered statistically significant.

## 3. Results

### 3.1. Characterization of Stem Cell Surface Markers of BMSCs

**Figure 1:**
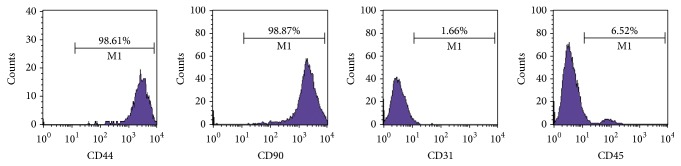
Characterization of BMSCs. Flow cytometry results of BMSCs at passage 3.

Cultured cells expressed CD44 (98.61%) and CD90 (98.87%) and did not express CD31 (1.66%) and CD45 (6.52%), demonstrating that the cells obtained from SD rats were BMSCs (Figure 1).

### 3.2. BMSCs Morphology, Viability, and Proliferation under NWPT

**Figure 2:**
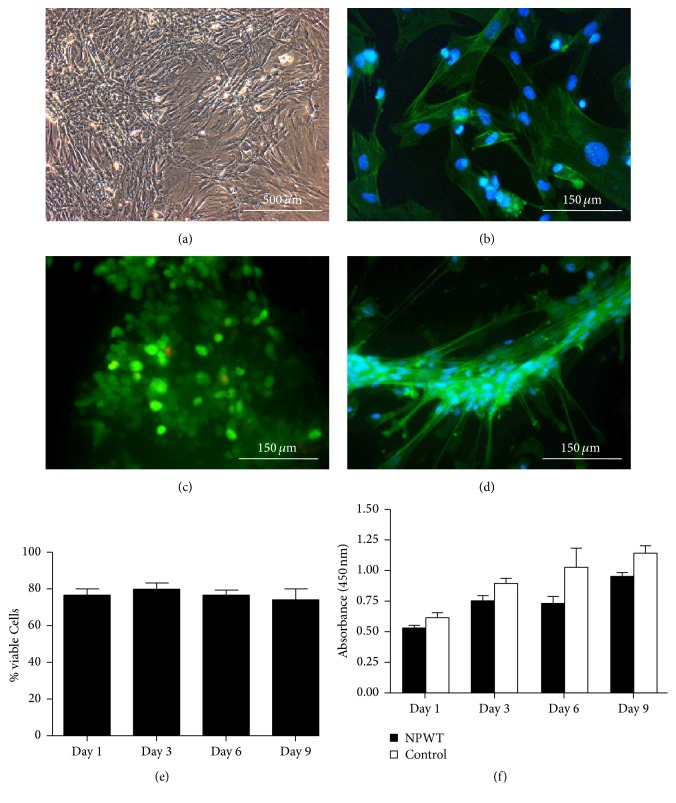
BMSCs morphology, viability, and proliferation under NWPT. (a) Light microscopy revealed morphology of BMSCs cultured in plates at 3 passage. (b) Fluorescent micrograph of BMSCs cultured in plates at 3 passages. (c) A representative image of live/dead assay at 9 days. Green: live cells, red: dead cells. (d) Fluorescent microscope image when BMSCs were cultured under NPWT. (e) The result of live/dead assay of BMSCs cultured under NPWT in vitro for 9 days. (f) An CCK-8 assay was used to assess proliferation of BMSCs with or without NPWT over 9 days. Data is given as the mean ± SD.

In comparison with standard culture (Figures 2(a) and 2(b)), BMSCs exhibited a spindle-shaped morphology under NPWT (Figure 2(d)). Of note, BMSCs cultured under NPWT maintained well viability (more than 76%) for up to 9 days (Figures 2(c) and 2(e)). More importantly, BMSCs under NPWT exhibited slightly increased proliferation over seven days (Figure 2(f)).

### 3.3. Induction of BMSC Differentiation by NPWT

**Figure 3:**
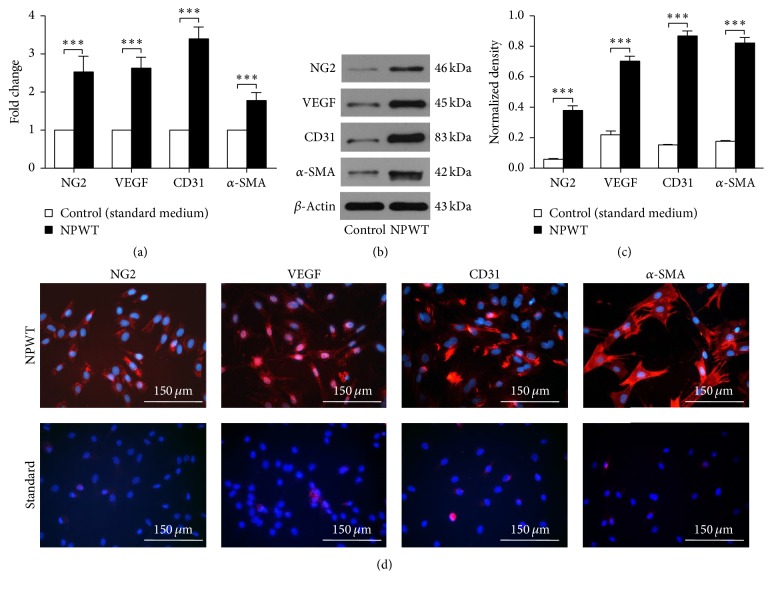
Effect of NPWT on BMSC differentiation. (a) qRT-PCR analysis of NG2, VEGF, CD31, and *α*-SMA gene expression under NPWT versus 2D culture with standard medium. The data of 2D culture were considered as 1. (b) Western blotting of NG2, VEGF, CD31, and *α*-SMA protein expression under NPWT versus 2D culture. (c) Quantification of western blotting. (d) Immunofluorescent staining demonstrated that BMSCs cultured under NPWT exhibited differentiated states compared with 2D standard. Data is given as the mean ± SD, ^*∗∗∗*^*p* < 0.001.

We examined different angiogenesis related cell markers including NG2 (for pericytes) and *α*-SMA (for smooth muscle cells, SMCs); CD31 and VEGF (for endothelial cells, ECs) to represent the differentiation state of BMSCs. After 9 days of NPWT, the western blot results showed increased protein expression compared with the control group for NG2 (0.3767 ± 0.0328, *p* < 0.001), VEGF (0.7020 ± 0.0344, *p* < 0.001), CD31 (0.8663 ± 0.0352, *p* < 0.001), and *α*-SMA (0.8211 ± 0.0351, *p* < 0.001) (Figures 3(b) and 3(c)). Similarly, real-time qPCR analysis and immunofluorescence staining confirmed the same results as those observed in western blotting analyses (Figures 3(a) and 3(d)).

### 3.4. Wound Healing

**Figure 4:**
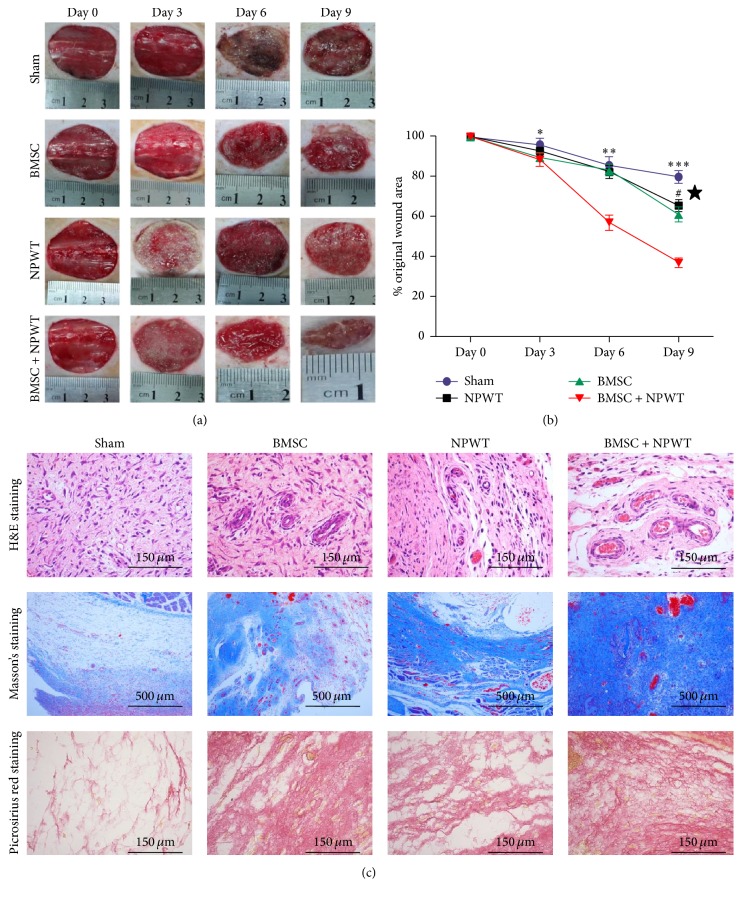
Evaluation of wound tissues. (a) Representative gross photos of the wounds treated with sham operation, BMSCs, NPWT, and BMSCs + NPWT. (b) Wound closure curves demonstrated significantly accelerated wound healing in BMSC + NPWT group. (c) H&E staining, Masson's trichrome staining, and picrosirius red staining of wounds at day 9. Data is given as the mean ± SD, ^*∗*^*p* < 0.05, ^*∗∗*^*p* < 0.01, and ^*∗∗∗*^*p* < 0.001 untreated versus BMSC + NPWT at different time points, respectively; ^#^*p* < 0.01 NPWT versus BMSC + NPWT; ^★^*p* < 0.001 local BMSC injection versus BMSC + NPWT.

Gross photographs of wound healing progression from each group in the cutaneous wound model were shown in Figure 4(a). Notably, wounds treated with BMSCs + NPWT showed accelerated healing at all time points compared with other treatment groups (Figure 4(b)). Specifically, there was remarkable acceleration of wound healing on day 9 in BMSC + NPWT group (37.15 ± 2.37%, *p* < 0.001) compared with untreated wounds (80.07 ± 3.16%).

As shown in Figure 4(c), by using H&E and Masson's and picrosirius red staining, wounds treated with BMSC + NPWT exhibited abundant blood vessel distribution and increased collagen fibers aggregation in a regular arrangement, as compared with the local BMSC injection, NPWT, and sham groups.

### 3.5. Wound Vascularization

**Figure 5:**
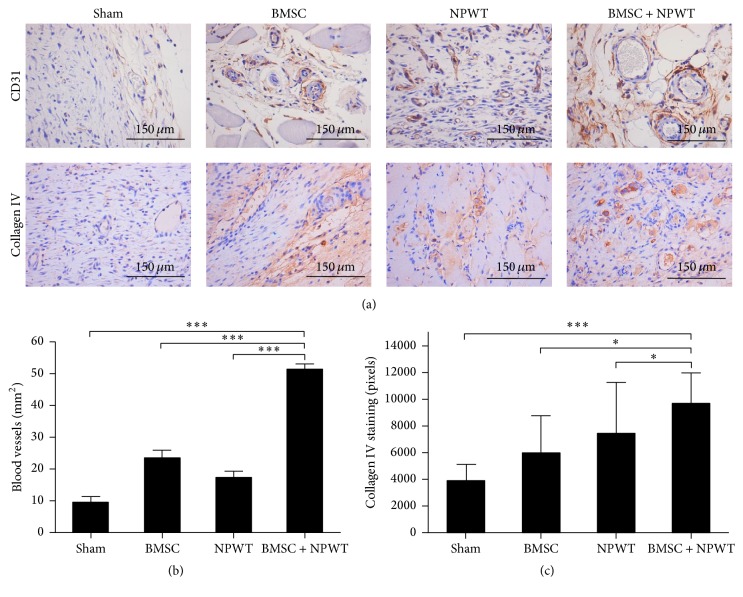
BMSC + NPWT accelerate the formation of vascularized granulation tissue. (a) Immunohistochemical staining for CD31 and collagen IV, representing newly formed blood vessels. (b) Quantification of newly formed blood vessels. (c) Quantification of collagen IV expression. Data is given as the mean ± SD, ^*∗*^*p* < 0.05, ^*∗∗∗*^*p* < 0.001.

Using immunohistochemistry to explore CD31 expression, we observed abundant new blood vessels in BMSC + NPWT treated wounds but few in the sham group wounds on day 9 (51.26 ± 1.644 vessels per mm^2^ versus 9.533 ± 1.752 vessels per mm^2^, *p* < 0.001) (Figures 5(a) and 5(b)). Using immunohistochemistry to explore collagen IV expression, a well-developed vascular network was also present in the BMSC + NPWT-treated wounds at day 9, whereas it was almost completely absent in sham group (Figures 5(a) and 5(c)).

**Figure 6:**
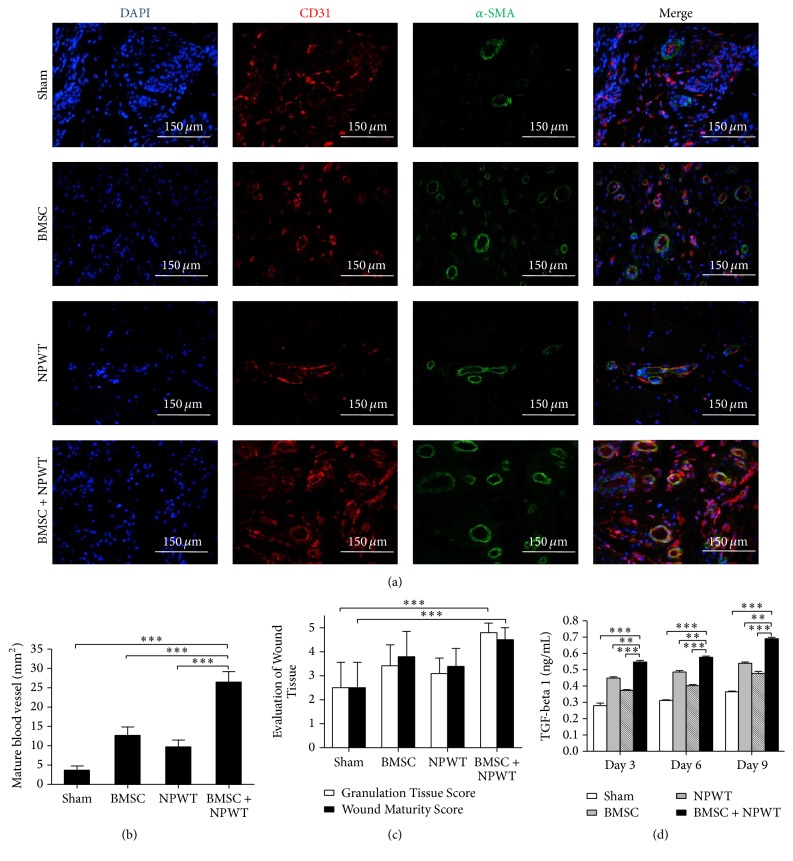
BMSC + NPWT accelerate the formation of mature vessel. (a) Immunofluorescence staining for CD31 and a-SMA. Red and green costaining represented mature blood vessels. Nuclei were stained with DAPI (blue). (b) Quantification of mature blood vessels. (c) Granulation tissue score and wound maturity score. (d) Concentration of TGF-*β*_1_ within wounds among different groups. Data is given as the mean ± SD, ^*∗∗*^*p* < 0.01, ^*∗∗∗*^*p* < 0.001.

Immunofluorescence costaining of CD31 and *α*-SMA representing mature blood vessels showed that BMSC + NPWT improved blood vessel maturity (26.433 ± 2.741 vessels per mm^2^) compared with sham group (3.530 ± 1.741 vessels per mm^2^, *p* < 0.001) (Figures 6(a) and 6(b)).

In addition, granulation and wound maturity scores in the BMSC + NPWT group were both significantly higher than those in the sham groups (*p* < 0.001) (Figure 6(c)). As a primary driving factor responsible for angiogenesis process during granulation tissue formation, TGF-*β*_1_ levels at all postwounding time points in wounds treated with BMSC + NPWT showed significantly higher concentrations than did untreated wounds (*p* < 0.001) (Figure 6(d)).

**Figure 7:**
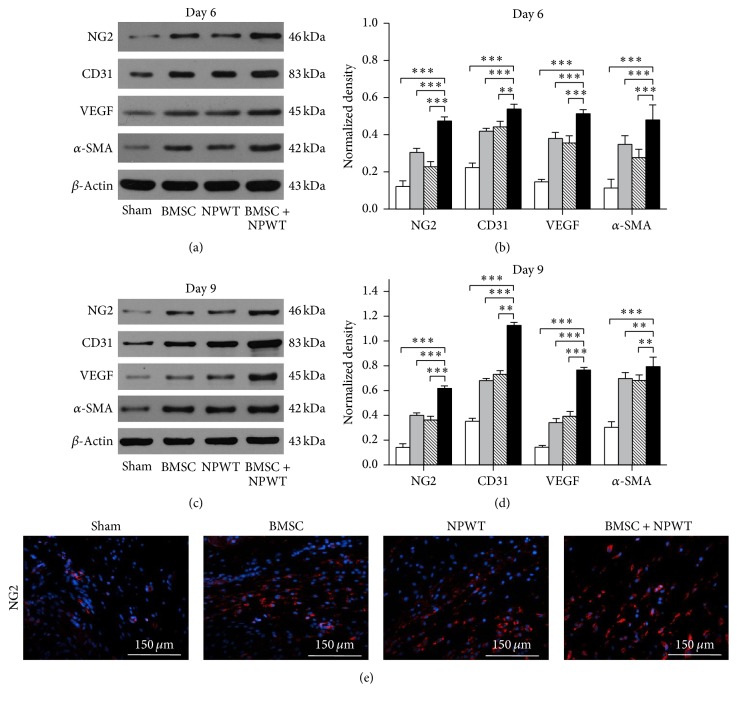
Assessment of angiogenesis related factors and cytokines. (a) Western blotting of NG2, VEGF, CD31, and *α*-SMA protein expression within wounds among different groups on day 6. (b) Quantification of western blot results on day 6. (c) Western blotting of NG2, VEGF, CD31, and *α*-SMA protein expression within wounds among different groups on day 9. (d) Quantification of western blot results on day 9. (e) Immunofluorescence staining of NG2 within wounds on day 9. Nuclei were stained with DAPI (blue). Data is given as the mean ± SD, ^*∗∗*^*p* < 0.01, ^*∗∗∗*^*p* < 0.001.

**Figure 8:**
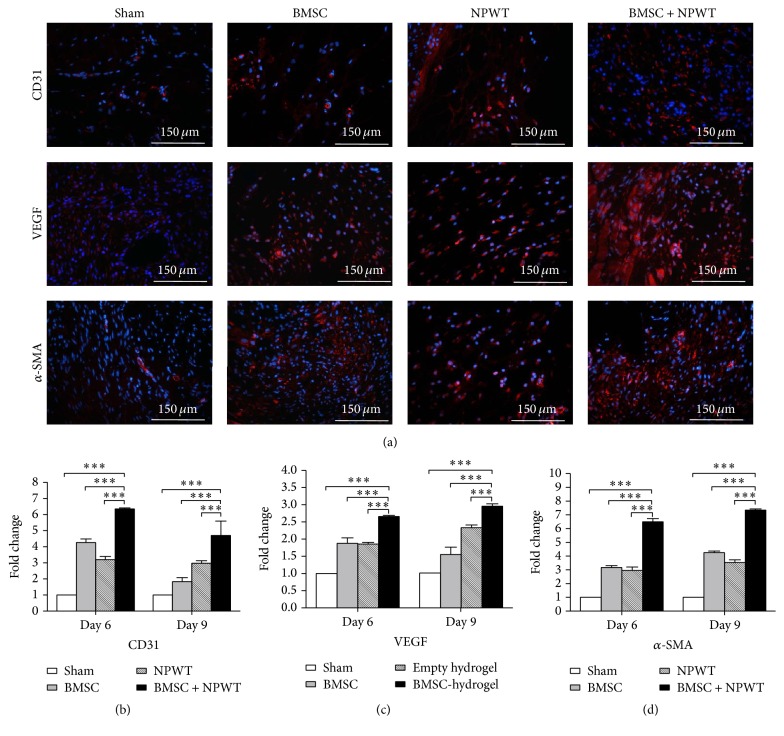
Assessment of angiogenesis related factors and cytokines. (a) Immunofluorescence staining of CD31, VEGF, and *α*-SMA within wounds among different groups on day 9. (b) RT-PCR results revealed more expression of CD31, VEGF, and *α*-SMA in wounds treated with BMSC + NPWT group compared to other three groups. Data is given as the mean ± SD, ^*∗∗∗*^*p* < 0.001.

In addition, western blot analyses reflected enhanced NG2, CD31, VEGF, and *α*-SMA protein levels in day 6 and 9 wounds treated with BMSC + NPWT compared with untreated controls (*p* < 0.001) (Figures 7(a), 7(b), 7(c), and 7(d)). Real-time-PCR analysis and immunofluorescence staining also reflected the same tendency (Figures 7(e), 8(a), 8(b), 8(c), and 8(d)).

## 4. Discussion

Our study investigated whether the combinatory strategy using BMSC injection coupled with NPWT can exert therapeutic effects in a murine cutaneous defect model and explored the underlying mechanisms involved. Our results demonstrated for the first time that BMSC + NPWT could significantly promote cutaneous wound healing, characterized by robust and enhanced vascularization at wound sites. Specifically, we have demonstrated that full thickness skin wounds treated with BMSCs combined with NPWT lead to acceleration of angiogenesis when compared to rats receiving topical administration of NPWT without stem cells. More importantly, we found that NPWT provided a beneficial microenvironment supporting better BMSC's viability and promoted their angiogenic capacity for abundant neoangiogenesis and maturation of blood vessels, suggesting that this strategy may serve as an alternative to aid fundamental soft tissue reconstruction for wound healing. Thus, a major challenge in regenerative medicine to create a functional microenvironment which supports and facilitates the angiogenic properties of BMSCs to enhance wound healing has been met, and this would further help to pave the way to clinical application.

During the past few years, negative pressure wound therapy (NPWT) has emerged as a treatment for those complex wounds that need effective therapy to heal. This method is the delivery of intermittent or continuous subatmospheric pressure through a particular pump, connecting to a poriferous and foam-surface dressing covered with an adhesive drape to maintain a vacuum environment [24]. Clinicians have consented that NPWT could stimulate a robust granulation tissue response compared with other available therapies [25–27]. But recently some different voice emerged, demonstrating that NPWT may prolong the healing time to wound closure and cause an increased need for skin grafts for fasciotomy wounds [28]. Another experimental study demonstrated that there was a detrimental effect exerted by NPWT on the extent of muscle fiber regeneration on a pig model [29]. Therefore, a complementary therapy that can compensate the drawback of NPWT is worth evaluating. Mesenchymal stem cells (MSC), which are recognized as pluripotent progenitor cells, have been proven to enhance tissue regeneration in several studies [30–32]. The effect of MSC transplantation to improve wound healing could be attributed to the inductive cell differentiation and the release of paracrine factors from them. However, the poor viability of the engrafted MSC at the recipient site often impedes their therapeutic application. As a result of that, it is significant to increase the survival of transplanted MSC and enhance their biological functions such as secretion of factors.

Previously, we have demonstrated that negative pressure (−125 mmHg) could induce the differentiation of periosteum-derived mesenchymal stem cells (P-MSCs) toward the osteogenic phenotypes after the 7 days cultured with the help of osteogenic inductive medium. For healing cutaneous wounds, some reports have been demonstrated that high levels of negative pressure (approximately −150–200 mmHg) may be efficient to induce macromechanical deformation of wounds and may be more favorable in soft tissue wounds [33, 34]. Therefore, we used −150 mmHg as the applied pressure in this study and proved that NPWT could induce BMSCs to differentiate into angiogensis related cells including pericytes, endothelial cells, and vessel smooth muscle cells, which are beneficial for the formation of granulation tissue in the absence of inductive medium. We hypothesized that this inductive therapeutic effect is due to the changed BMSCs' morphology with thicker appearance and more organized actin cytoskeleton caused by NPWT. In the vitro experiment, BMSCs exhibited a spindle-shaped morphology and stretched cytoskeleton under NPWT, which is consistent with the previous study [19]. This is a crucial finding because frequently there is a lack of sufficient stimulation within the wounds of patients to induce the transplanted BMSCs to differentiate into the desired cell types.

Particularly, hypoxia condition is beneficial for maintaining hMSCs in proliferating condition, especially in the late stage of hypoxia microenvironment such as 7 days. In addition, hMSCs would maintain their growth-rates even after reaching confluence and also exhibit differences in the cell and nuclear morphologies as well as accelerated ECM organization, leading to the better tissue regeneration [22]. Therefore, an optimized therapy that may serve as a stimulus to generate hypoxia condition and microenvironment would be helpful to enhance the viability of transplanted BMSCs. Erba et al. [21] have demonstrated that the NPWT treatment accelerates wound repair not only by a hypoxia driven well organized new vessels within the wound bed, but also by mechanical forces mediated activation of angiogenesis related cells proliferation. They concluded that the subatmospheric pressure wound therapy was capable of establishing a gradient hypoxia environment within wounds. In our study, both of in vitro and in vivo experiment showed enhanced activity of BMSCs under NPWT compared with transplanting cells alone, which supported the previous finding. Hence, we hypothesise that NPWT could generate a relative hypoxia microenvironment, stimulating transplanted BMSCs to maintain a better activity, leading to enhanced viability of the cells for further cutaneous repair.

Wound healing is a dynamic development that comprises the network among the extracellular matrix (ECM), growth factors, and various types of resident cells. It begins with the proliferation of fibroblasts generation of collagen fibers and angiogenesis, leading to granulation tissue, wound contraction, and epithelialization. Notably, vasculogenesis and angiogenesis are indispensable for wound healing. Angiogenesis consists of the sprouting and remodeling of the primitive blood vessels, followed by the stabilization of mural cells (e.g., pericytes for small vessels and smooth muscle cells for large vessels) [3]. The interaction of ECs and mural cells aids to stabilizing the microvessels, which is required for vessel maturation [35]. Therefore, the pericytes, ECs, and smooth muscle cells play significant roles in angiogenesis. In our study, the in vitro and the in vivo tests showed BMSC + NPWT enhanced the expression of NG2, CD31, VEGF, and *α*-SMA, representing the activity of pericytes, ECs, and smooth muscle cells, respectively. Furthermore, we performed the immunohistochemical staining of CD31, a membrane-spanning protein expressed early in blood vessel development, to assess the newly formed vessels, and collagen IV, which plays an important role in ECM formation for wound healing [36]. Besides, the generation of mature blood vessels is characterized by the endothelial cells completely encircled by smooth muscle cells, which can be showed by CD31 and *α*-SMA, respectively [37]. Hence, the costaining of CD31 and *α*-SMA can be utilized to locate the mature blood vessels. The results of immunohistochemistry and immunofluorescence staining proved that treatment of the skin defects with BMSC + NPWT not only enhanced the number of newly formed blood vessels but also improved their maturity (Figures 5 and 6). Coupled with the western blotting and PCR results indicating the upregulated expression of NG2, CD31, VEGF, and *α*-SMA (Figures 6 and 7), our findings demonstrated that BMSC + NPWT could enhance the BMSC angiogenic capacity, resulting in accelerated wound healing and regeneration.

Currently, studies of cell-based treatment of complex wounds have largely focused on the use of MSCs transplanted topically, via injection around the edges of the wound directly [38, 39]. The experimental study performed on rodent wound models and clinical trials of chronic wounds has demonstrated that MSCs can effectively and safely accelerate wound healing [40]. Since the isolation and propagation of MSCs is a relative simple process, this therapy is particularly of interest [41]. Additionally, the use of MSCs is with low clinical risk. For NPWT, it is widely used in wound care in adults, and there are even numbers of peer-reviewed case studies in pediatrics, reflecting its safety and effectiveness in the pediatric population [42]. Hence, the process using BMSCs and NPWT as a combinatory treatment would be feasible and simple on patients. Particularly, our findings can assist clinicians to treat the complex and chronic wounds with an alternative method instead of using traditional way such as changing gauze, which is time-saving and economical, eliminating the risk of potential immunogenicity risk and infection.

## 5. Conclusion

These findings demonstrate that the capacity of NPWT to enhance BMSC angiogenic property for cutaneous wound healing potentially by preserving the viability of the cells, stimulating, and inducing them to differentiate into the desired cells that are beneficial for angiogensis within wounds. This combinatory strategy is superior to either of cells transplantation and NPWT therapy alone, leading to the accelerated wound healing. Further optimization of the applicable parameter of NPWT and BMSCs may provide more benefits to clinical application.

## Figures and Tables

**Table 1:** Criteria for histological scores of wounds. Criteria of scoring system for granulation tissue formation and wound maturity.

Score	Granulation tissue formation	Wound maturity
(1)	No/minimal granulation tissue	Limited cells present or highly inflammatory
(2)	Low granulation tissue	Predominantly inflammatory
(3)	Moderate granulation tissue	Equivalence between inflammatory and proliferative
(4)	Extensive granulation tissue	Predominantly proliferative
(5)	Very extensive granulation tissue	Highly proliferative

**Table 2:** The primer sequences for each primer used in the real-time RT-PCR. The primer sequences and annealing temperature of CD31, *α*-SMA, VEGF, NG2, and *β*-actin.

Genes	Primer sequences	Annealing temperature (°C)
*β*-Actin	Forward: CGTTGACATCCGTAAAGACCTC Reverse: TAGGAGCCAGGGCAGTAATCT	58
CD31	Forward: GATCTCCATCCTGTCGGGTAAC Reverse: GTGTCATTCACGGTTTCTTCGT	58
*α*-SMA	Forward: CAACCCCTATACAACCATCACAC Reverse: CCCAAACTGCTTGCGTAACC	58
VEGF	Forward: ATCTTCAAGCCGTCCTGTGTG Reverse: AGGTTTGATCCGCATGATCTG	58
NG2	Forward: TTACCTTGGCCTTGTTGGTC Reverse: GATGATCTGTTTGGCCTGCT	58
